# Supplementary material for: Structure of Blm10:13S proteasome intermediate reveals parallel assembly pathways for the proteasome core particle
Source: bioRxiv. 2024 Nov 5:2024.11.04.621988. Preprint. [Version 1] doi: 10.1101/2024.11.04.621988 (PMC11580919; doi:10.1101/2024.11.04.621988)
Supplement: Supplement 1 [file NIHPP2024.11.04.621988v1-supplement-1.pdf]

bioRxiv preprint doi: https://doi.org/10.1101/2024.11.04.621988; this version posted November 5, 2024. The copyright holder for this preprint (which was not certified by peer review) is the author/funder, who has granted bioRxiv a license to display the preprint in perpetuity. It is made available under aCC-BY 4.0 International license.

Supplementary Table 1. Strain list

Strain	Genes manipulated	Repair DNA	Fig.	Ref.
sUB61	*MATα lys2-801 leu2-3, 2-112 ura3-52 his3D200 trp1-1*	n/a	7,8b-c, 6S	(a)
sJR785	*MAT A ump1::UMP1-CBP-TEV-ZZ-(His3MX6)*	n/a	7,8b-c, 6S	(b)
sJR792	*MAT A pba1::PBA1 (NAT) ump1::UMP1- CBP-TEV-ZZ-(His3MX6)*	n/a	8c	(c)
sJR793	*MAT A pba1::PBA1 (HYG) blm10::BLM10 (NAT) ump1::UMP1- CBPTEV-ZZ-(His3MX6)*	n/a	8c	(c)
sJR794	*MAT A blm10::BLM10 (NAT) ump1::UMP1- CBPTEV-ZZ-(His3MX6)*	n/a	8c	(c)
sJR795	*MAT A pba1::PBA1Δ3C (KAN) ump1::UMP1- CBPTEV-ZZ-(His3MX6)*	n/a	7	(c)
sJR809	*MAT A pba1::PBA1 Δ3C (KAN) pba2:: PBA2 Δ3C (NAT) ump1::UMP1- CBPTEV-ZZ-(His3MX6)*	n/a	7	(c)
sJR2578	*MAT A pba1::PBA1*^{L2S}*Δ3-17 ump1::UMP1-CBP-TEV-ZZ-(His3MX6)*	pRL1478	7	This study
sJR2583	*MAT A pba1::PBA1* ^{L2S}*Δ3-31 ump1::UMP1-CBP-TEV-ZZ-(His3MX6)*	pRL1503	7	This study
sJR2584	*MAT A pba1::PBA1* ^{L2S}*Δ3-31 pba1::PBA1Δ3C (KAN) ump1::UMP1-CBP-TEV-ZZ-(His3MX6)*	pRL1503	7	This study
sJR2586	*MAT A pba1::PBA1* ^{L2S}*Δ3-31 pba1::PBA1Δ3C (KAN) pba2:: PBA2 Δ3C (NAT) ump1::UMP1-CBP-TEV-ZZ-(His3MX6)*	pRL1503	7	This study
sJR2588	*MAT A pba1::PBA1*^{L2S}*Δ3-17 blm10::GPDpGFPBLM10 (CloNAT) ump1::UMP1-CBP-TEV-ZZ-(His3MX6)*	pRL1478	7	This study
sJR2589	*MAT A pba1::PBA1* ^{L2S}*Δ3-31 blm10::GPDpGFPBLM10 (CloNAT) ump1::UMP1-CBP-TEV-ZZ-(His3MX6)*	pRL1503	7	This study
sJR1012	*MAT A blm10::GPDpGFPBLM10 (NAT) ump1::UMP1-CBP-TEV-ZZ-(His3MX6)*	n/a	7	(d)
sJR2614	*MAT A pba4::PBA4-CBP-TEV-ZZ-(His3MX6)*	n/a	8b	(b)
sJR2543	*MAT A ump1::UMP1*^{R93A} *ump1::UMP1- CBPTEV-ZZ-(His3MX6)*	pRL1470	6S	This study
sJR2545	*MAT A ump1::UMP1*$^{R93A,\ G89A}$ *ump1::UMP1- CBPTEV-ZZ-(His3MX6)*	pRL1471	6S	This study

genotype is: ***MAT A his3**Δ0 leuΔ0 met15Δ0 ura3Δ0,* except for sUB61 which is *MATα lys2-801 leu2-3, 2-112 ura3-52 his3D200 trp1-1*. (a) Finley, D., Ozkaynak, E. & Varshavsky, A. **1987** Cell 48, 1035-1046. (b) Ghaemmaghami, S. et al. **2003** Nature 425, 737-741. (c) Wani, P. et al. **2015** Nature Communications 7384, 1-11. (d) Burris, A. et al 2021 Journal of Biological Chemistry, 296, 1-18.

bioRxiv preprint doi: https://doi.org/10.1101/2024.11.04.621988; this version posted November 5, 2024. The copyright holder for this preprint (which was not certified by peer review) is the author/funder, who has granted bioRxiv a license to display the preprint in perpetuity. It is made available under aCC-BY 4.0 International license.

Supplementary Table 2. Crispr/Cas9 gRNA and repair DNA

Target gene	gRNA	Plasmid with gRNA & Cas9	Repair DNA	Sequence repair DNA
UMP1	TACTACCGTTTGTTGAAAGG	pJR1162	pRL1470	AGCAAGAAGGCGGTGCAGTACCTCTTTCCACACAATTGAATGACAGACATCCATTAGAATCAACATTGAAGAACTGGGAAACCACACAGCGCCAAAGACAAATGGAACAGTATCGACAAATATTTGGCATTGCCGAACCAATGAAgAGaACtATGGAgATGGAAATCGTCAATgcaACCGACTTCAAtCCaCTgTCtACcAACGGTAGTATACACCGTGACATACTACTGAACAAAGAGTGCAGCATCGATTGGGAGGACGTCTACCCTGGTACTGGCCTACAAGCCAGCACCATGGTAGGTGATGACGTCCACAGC
UMP1	TACTACCGTTTGTTGAAAGG	pJR1162	pRL1471	AGCAAGAAGGCGGTGCAGTACCTCTTTCCACACAATTGAATGACAGACATCCATTAGAATCAACATTGAAGAACTGGGAAACCACACAGCGCCAAAGACAAATGGAACAGTATCGACAAATATTTGGCATTGCCGAACCAATGAAgAGaACtATGGAgATGGcAATCGTCAATgcaACCGACTTCAAtCCaCTgTCtACcAACGGTAGTATACACCGTGACATACTACTGAACAAAGAGTGCAGCATCGATTGGGAGGACGTCTACCCTGGTACTGGCCTACAAGCCAGCACCATGGTAGGTGATGACGTCCACAGC
PBA1	CTACTCTAGCAAAATATGAT	pJR1164	pRL1478	TTGGCACCTAATTCAGACATTTTTGCTTTTGTTCCTCCACTCAAATGTAGTCTTTTTCAATTCTGAAGTGGGATTATTTATAGATGACGTTTGACTTGATGAAGTTTTATTGCCTTTACTTTTTTTTTTTCTTTCGCATCAAAATTTCAGACTTCATTGAAATCAATTTAAGAAGTTGCGTGGCAATAGGTGGCAACAGGCGAAATAATAAGAAACAGCGACCAAGATATCATCGCACTACAGTAAAATTTTCATTTATAGCGATGagtCTCCCAGAGATTTCAAAAAACCTGCAATCTTTAGAAGTCTGCCCGGTTCCAAAAGTTGAGTTTCCTCAGGACTTGGATGTT
PBA1	CTACTCTAGCAAAATATGAT	pJR1164	pRL1503	TTGGCACCTAATTCAGACATTTTTGCTTTTGTTCCTCCACTCAAATGTAGTCTTTTTCAATTCTGAAGTGGGATTATTTATAGATGACGTTTGACTTGATGAAGTTTTATTGCCTTTACTTTTTTTTTTTCTTTCGCATCAAAATTTCAGACTTCATTGAAATCAATTTAAGAAGTTGCGTGGCAATAGGTGGCAACAGGCGAAATAATAAGAAACAGCGACCAAGATATCATCGCACTACAGTAAAATTTTCATTTATAGCGATGagtCCGGTTCCAAAAGTTGAGTTTCCTCAGGACTTGGATGTTCCTCAATATTCGACAGCCGTCATTACTACTAAGATAATGAACCCATTATTCCCCAAAAATCTGCTTCAATTAACCTCTATTGGTGAAATTAAAACTACATTGACGGTGAAAAGTCCAAGTCTTCCCCAATCTTCAGGTAAACACTCGTGG
